# Community Health Workers in Pandemics: Evidence and Investment Implications

**DOI:** 10.9745/GHSP-D-21-00648

**Published:** 2022-04-28

**Authors:** Madeleine Ballard, Ari Johnson, Iris Mwanza, Hope Ngwira, Jennifer Schechter, Margaret Odera, Dickson Nansima Mbewe, Roseline Moenga, Prossy Muyingo, Ramatulai Jalloh, John Wabwire, Angela Gichaga, Nandini Choudhury, Duncan Maru, Pauline Keronyai, Carey Westgate, Sabitri Sapkota, Helen Elizabeth Olsen, Kyle Muther, Stephanie Rapp, Mallika Raghavan, Kim Lipman-White, Matthew French, Harriet Napier, Lyudmila Nepomnyashchiy

**Affiliations:** a Community Health Impact Coalition, London, United Kingdom.; b Icahn School of Medicine at Mount Sinai, New York, NY, USA.; c Muso, Bamako, Mali.; d Community Health Roadmap, New York, NY, USA.; e VillageReach, Lilongwe, Malawi.; f Integrate Health, New York, NY, USA.; g Ministry of Public Health, Nairobi, Kenya.; h Ministry of Health, Kasungu District, Malawi.; i Living Goods, Kampala, Uganda.; j Ministry of Health, Kono District, Sierra Leone.; k Financing Alliance for Health, Nairobi, Kenya.; l Arnhold Institute for Global Health, Icahn School of Medicine at Mount Sinai, New York, NY, USA.; m Nama Wellness, Mukono, Uganda.; n Community Health Impact Coalition, New York, NY, USA.; o Possible, Kathmandu, Nepal.; p Medic, San Francisco, CA, USA.; q Last Mile Health, New York, NY, USA.; r Muso, Abidjan, Cote d'Ivoire.; s Possible, New York, NY, USA.; t Clinton Health Access Initiative, Salt Lake City, UT, USA.; u Community Health Acceleration Partnership, New York, NY, USA.

## Abstract

Community health workers have long played a critical role in preventing, detecting, and responding to pandemics across the globe. To expand, improve, and institutionalize these services, changes in the approach to bi/multilateral aid and private philanthropic investments in low- and middle-income countries are required.

## Key Messages

- A strong and accessible national health system, including at the community level, is critical for pandemic preparedness and response.
- Community health workers who are equipped, trained, and paid as part of a well-functioning health system can help prevent epidemics from becoming pandemics and maintain health care delivery amid significant disruption.
- To achieve resilient health systems, bi/multilateral aid and private philanthropies need to review their investment practices to replace those that cause harm (high transaction costs, earmarking, short-termism, appropriation of sovereignty) with practices that ensure timely and effective implementation of priorities set by government stakeholders (pooling, longer commitments, and alignment with evidence-based guidelines)

## INTRODUCTION

Community health workers (CHWs)—people trained to meet the health needs of their communities by delivering care in their communities—have been a critical part of health care delivery across diverse contexts for over a century.^1^ They have also been woefully under-supported: recent estimates suggest that across the African continent, more than 4 in 5 CHWs are unpaid.^2^ In the context of increasing global health insecurity and a burgeoning health workforce crisis, this trend must change. The coronavirus disease (COVID-19) pandemic reminds us that CHWs who are equipped, trained, and paid as part of a well-functioning health system can help keep pandemics in check and maintain health services equity and access.

True pandemic preparedness and response requires replacing bi/multilateral aid and private philanthropic investments that hinder CHW institutionalization and professionalization (high transaction costs, earmarking, short-termism, appropriation of sovereignty) with investments made in partnership with a recipient country. In particular, these funds should be deployed rapidly and flexibly against government-set priorities.

In this commentary, we review the critical roles of CHWs during pandemics and discuss how rethinking common bi/multilateral aid and private philanthropic investment practices can help create resilient health systems everywhere.

## CHWS ARE CRITICAL TO KEEPING PANDEMICS IN CHECK

CHWs—if equipped with ongoing training, supervision, remuneration, medical commodities, and personal protective equipment (PPE)—can prevent, detect, and respond to pandemics by carrying out tasks as diverse as leading community-based infection prevention and control, facilitating safe sample collection, conducting contact tracing, accelerating vaccination roll-out, and providing home-based care to those in quarantine (Table 1).^3^^–^^20^

**TABLE 1.** Examples of How CHWs Have Prevented, Detected, and Responded to Pandemics

	Intervention	Example^a^
**Prevent**	**Educate communities regarding signs, symptoms, and transmission routes.** Lead skill building for personal preventive measures such as social distancing, hand hygiene, coughing/sneezing into elbows, and water, sanitation, and hygiene interventions. Address public health misinformation as trusted messengers during in-person interactions.	**DRC, Liberia:** During the 2014–2016 Ebola epidemic, CHWs were part of the interdisciplinary teams of nurses, doctors, and other health workers who played a critical role in reducing transmission through promoting social distancing and other infection prevention and control measures.^3^ **Global:** By April 2020, CHWs reached 2.5 million households across 27 countries as part of countries' national strategies during the COVID-19 epidemic.^4^
	**Supply: Support, lead, or reinforce community and facility-based infection prevention and control measures**, such as construction of triage areas, use of personal protective equipment (e.g., face masks and gloves), and creation of hand hygiene stations.^5^ Distribute PPE to the community.	**Cote d'Ivoire** employed CHWs during the 2014–2016 Ebola epidemic for disease prevention and control as did **Liberia, Sierra Leone, and DRC**.^6^
	**Vaccinate: CHWs have a critical role at every stage of vaccine rollout:** planning; identifying target populations; outreach, engagement, and education; mobilization; and tracking and reporting outcomes.^7^ Critically, this requires that CHWs be protected with PPE and vaccinated as part of the initial allocation for health workers.	**Ethiopia, India, Malawi, Pakistan:** Health extension workers, accredited social health activists, HSAs, and lady health workers routinely provide intramuscular medicines.^8^^,^^9^ HSAs currently serve as COVID-19 vaccinators in some parts of Malawi.
**Detect**	**Detect signs and symptoms** in community members and **conduct rapid tests**, where available.^10^	**South Africa** deployed 28,000 CHWs to screen more than 7 million people for COVID-19.^11^
	**Facilitate safe sample collection** in communities and health facilities of samples and rapid transport to laboratories for analysis, thus reducing risks of nosocomial transmission.	**Sierra Leone:** As part of a post-Ebola laboratory strengthening, CHWs were enlisted to coordinate the collection and transport of sputum samples for tuberculosis testing from patients in the community to the laboratory.^12^
	**Alert** through integration into community events-based surveillance systems.^5^	**DRC:** During the 2014–2016 Ebola epidemic, CHWs filed alerts that were investigated within 24 hours.^13^ **Madagascar:** During the 2017 plague outbreak, 4400 CHWs and 340 supervisors were trained to conduct community-based active surveillance, contact tracing, and follow-up activities across the country.^14^ **Kenya:** During the COVID-19 pandemic, 6000+ CHWs were trained to use a mobile tool for community event-based surveillance, which was applied across 10 counties.^15^ Modeling indicates that CHWs participating in disease surveillance systems detect outbreaks to a comparable degree and with better timeliness compared to professional health care data entry staff.^16^
**Respond**	**Communicate rapidly and effectively** to residents in pandemic areas, including taking the time needed to communicate health information in a tailored and relevant way and combat the spread of misinformation.^17^	**Malawi, Togo:** As part of routine home visits, CHWs help track issues through community conversations and chats with informants, and tracking and media reporting, messaging, social media posts about COVID-19.
	**Enable self-isolation** and **Monitor patients** in the community while ensuring delivery of food, social, and medical support. Monitor patients for clinical deterioration and support rapid referral of individuals who require hospitalization, reinforcing links between the health system and communities.	**Kenya, India:** CHWs led home-based care for COVID-19 patients not requiring hospitalization.^18^^,^^19^
	**Conduct contact tracing**, symptom reporting, and monitoring of contacts of COVID-19 patients to ensure access to testing and treatment for those who develop signs and symptoms.^17^	**Nigeria:** CHWs' thorough knowledge of the landscape significantly shortened the commute time and led to more successful clinical outcomes. Their presence also served to facilitate community entry and acceptance among residents.^20^ **Africa CDC's** PACT initiative supported over 18,000 CHWs across 27 countries to conduct contact tracing and testing referrals. A recent evaluation of the PACT CHW program, based on feedback from 10 countries, suggests that it positively influenced the COVID-19 response and elevated the importance of CHWs' roles within primary care (Diana Nsubuga, PhD, email communication, July 28, 2021). As of August 2021, the 18,000 CHWs deployed supported 2.5 million household visits for risk communication and community engagement activities, active case search and contact tracing of more than 1.6 million contacts, and facilitation of testing referrals for 78% of suspect cases.

Abbreviations: CDC, Centers for Disease Control and Prevention; CHW, community health worker; COVID-19, coronavirus disease; DRC, Democratic Republic of the Congo; HSAs, health surveillance assistants; PACT, Prevention and Access to Care and Treatment; PPE, personal protective equipment.

a The authors of the article work predominantly in South Asia and Africa, from which the majority of the examples are drawn.

## CHWS MAINTAIN ESSENTIAL HEALTH SERVICES DURING PANDEMICS

Pandemics often precipitate declines in essential health service utilization, which can ultimately kill more people than the disease outbreak itself.^21^ CHWs—if equipped with ongoing training, supervision, remuneration, essential commodities, and personal protective equipment—are critical in maintaining equitable access to these services.

Large disruptions in health care utilization occurred as a result of the COVID-19 pandemic and endured for many months.^22^ How do we maintain essential health services in crisis and ensure strong systems are capable of responding to changing care-seeking behaviors as a result of fear of facility-based transmission?

It is important to note that CHWs in most countries provide not only promotive but also clinical care.^23^ Common tasks include Integrated Community Case Management (iCCM) (pneumonia, diarrhea, and malaria treatment), providing oral and injectable contraceptives, treating malnutrition, and administering vaccines for children. In many contexts, CHWs provide a significant proportion of primary care services delivered to uncomplicated febrile patients. In Liberia, for example, 45% of malaria cases are treated by CHWs; in Rwanda 56%.^24^^,^^25^ Such levels of contribution by CHWs have been noted in other countries.^26^

Recent evidence from 27 districts across 4 countries in sub-Saharan Africa (Kenya, Mali, Malawi, Uganda) found that CHWs supported in line with the WHO Guidelines (i.e., paid, equipped, and continuously trained) and protected with adequate PPE were able to maintain speed and coverage of community-delivered care during the pandemic.^27^ Specifically, there were no disruptions to the coverage of proactive household visits or iCCM assessments or to the speed with which iCCM was delivered, pregnancies were registered, and postnatal care was received. While this study did not look specifically at equity, previous studies have established that many community-oriented primary health care programs have an explicit equity promotion component,^28^ CHWs promote equitable access to promotive, preventive, and curative services at the household level,^29^ and CHW-delivered interventions (including home visits) improve equity in maternal and newborn health.^30^ Despite such high potential and powerful equity dividend, the majority of CHWs globally remain unpaid, without essential medications,^31^ inadequately supervised, and largely unsupported. This evidence suggests that the opportunity cost of not professionalizing CHWs may be substantially larger than previously estimated in light of the inevitability of future pandemics.^32^

Despite the high potential and powerful equity dividend, the majority of CHWs globally remain unpaid, without essential medications, inadequately supervised, and largely unsupported.

## THE BEST PANDEMIC RESPONSE IS A STRONG, ACCESSIBLE NATIONAL HEALTH SYSTEM

For CHWs to prevent, detect, and respond to pandemics and maintain delivery of essential health services in the context of significant disruption then, they must be treated as professionals. In other words, the best pandemic response is a strong, preexisting primary health care system integrated with the community level.

Two authors of this commentary work as CHWs in Kenya. They note the difference in how they were supported during the poliovirus 2 vaccination drive of May and July 2021^33^^,^^34^ compared to other times during the COVID-19 pandemic. During the polio campaign, they were integrated into national data systems, received consistent supportive supervision, and received prompt payment for their work. The campaign was a success. What possibilities await the world if a similar focus was applied to supporting CHWs in their work at all times?

By leveraging well-established, evidence-based tools such as the Community Health Worker Assessment and Improvement Matrix^35^ and World Health Organization (WHO) CHW Guidelines,^36^ governments can design and invest in high-performing CHW programs during and after this pandemic.

The following recommendations have a high potential for long-lasting impact.

### Include CHWs in National CHW Registries

Include CHWs in national, Ministry of Health (MOH)-governed human resources for health registries. CHWs cannot be supported to respond to pandemics or maintain essential services unless MOHs know who and where they are. Such registries are vital to support existing CHWs and to identify and close coverage gaps in pursuit of universal health coverage.^37^

### Provide Accreditation

Institutionalize minimum practice standards to ensure CHWs possess the knowledge and competencies required to deliver quality patient care, build trust, and help formally acknowledge the key role CHWs in many cases already play. As with all accredited professionals, CHWs must be meaningfully included in decision making and represented on bodies that plan their work and working conditions.

### Increase Accessibility

Ensure that care provided by CHWs is provided without charging point-of-care user fees, an approach that is proven to improve access to care and, therefore, equity.^29^

### Provide Ongoing Skill Development

Provide CHWs ongoing training in the essential clinical and nonclinical skills and knowledge needed to support comprehensive community health care (including education and provision of curative care).

### Equip CHWs With Adequate Supplies

Integrate CHW medical and nonmedical supply needs like PPE and essential medicines into national forecasting and supply chain processes (e.g., planning, quantification, distribution, and financing).

### Provide Supervision

Ensure all CHWs have a dedicated supervisor. This is critical during pandemics as professional protocols evolve and health and safety risks increase. Having supporting supervisors review summary statistics of CHW performance, assess patient experience, and support CHWs improves the use of community-based care and quality of health delivery.

### Offer Competitive Pay

Pay CHWs at a competitive rate relative to the respective market and pay them consistently, on time, and commensurate with the job. Give CHWs the benefits they deserve including hazard pay, family leave, compensation for overtime, and sick leave.

CHWs should be paid at a competitive rate relative to the respective market and paid consistently, on time, and commensurate with the job.

### Offer Opportunities for Advancement

Offer professional advancement opportunities, including the possibility of becoming a dedicated CHW supervisor. Include CHWs in decision making at all levels.

### Use Data for Performance Monitoring

Invest in comprehensive, nonvertical digital data systems, as well as training on data literacy to equip CHWs to document their visits consistently in a standardized format and report data to public sector monitoring and evaluation and logistics management information systems (e.g., health management information system). These data should be accessible to CHWs, their community, and their supervisor with tools for easy-to-understand data visualization and interpretation to inform performance management, public accountability, and robust monitoring and evaluation.

### Ensure a Manageable Workload

To achieve universal health coverage and avoid overburdening CHWs, it is critical to map CHW task allocation and time use.^38^

In summary, CHWs must be fully and formally integrated into the national health system to identify and treat cases, perform contact tracing, and refer patients to health facilities to receive timely clinical care. Investments in routine community health system strengthening form the underlying foundation of resilient health systems that can adequately respond to outbreaks and pandemics.

## INVESTMENT IMPLICATIONS: ENDING COVID-19 AND PREVENTING THE NEXT PANDEMIC

Global crises present opportunities for landmark policy changes. After World War II, Japan achieved universal health coverage, and after Ebola, Liberia launched a national cadre of paid, professionalized CHWs.

Responding to COVID-19 means addressing health crises, economic crises, and political crises at the same time. COVID-19 has exposed and exacerbated systemic inequalities, the sharp focus on which has diminished tolerance for inequality and catalyzed louder demands to dismantle inequitable systems. Multilateral, bilateral, and other donors have responded by increasing their investments in COVID-19 significantly, but not all investments in low- and middle-income countries are equal.

The best investment decisions are made in partnership with a recipient country and when funds can be deployed flexibly enough against government-set priorities as national health systems will outlast most civil society efforts. This is especially true through this pandemic where multiple shifts in priorities and quick decisions are needed to meet the challenges on the ground. MOHs can explain the complex funding landscape of multilateral, bilateral, philanthropic, and national investments; they will identify key gaps, funding priorities, and trusted implementing partners. Working with MOHs to strengthen community health provision ensures that we are all better prepared to face future pandemics. In addition, national civil society and local communities can play a key role in ensuring the equity, sustainability, and accountability in such partnerships.

Working with MOHs to strengthen community health provision ensures that we are all better prepared to face future pandemics.

One example of this type of government-led funding is the Community Health Roadmap Catalytic Fund, a unique fund designed to meet small-scale, flexible funding needs that are prioritized by community health coordinating mechanisms within MOHs.^39^ In this crisis, this fund supported the MOH COVID-19 response at the community level in Malawi and Zambia, getting funds to governments before large funding resources were available. Testimonials from MOH officials indicate this quick action helped both countries kickstart their response (Charles Mwansambo and Sylvia Chila, video communication, November 2020).

The best investments respond to the current crisis while also helping to build back better long-term through advancing CHW professionalization. An example of this is the COVID-19 Action Fund for Africa (CAF-Africa). By integrating with national COVID-19 responses, this collaboration was able to provide PPE to CHWs in a moment of acute need and also contribute to recognition, equality, and pay for CHWs across the continent.^40^ In Uganda, for instance, the government announced plans to compensate CHWs, known locally as village health teams, for their role in combating COVID-19 shortly after receiving a shipment of PPE. One official described this change as “a dream for the Ministry of Health for the last 20 years”—reinforcing the notion that crises present opportunities for landmark policy change.^41^

## IDEAS FOR ACTION

As CHWs and allies with a vested interest and shared commitment toward professionalization of CHWs and who have witnessed firsthand the detrimental practices that hold back community-delivered care, we offer several considerations to funders that share our interest in redressing systemic issues and propelling community health systems into a new era of achieving their full potential. New philanthropic investments should align with and support government strategies according to their needs and changing circumstances, including the demands of civil society. Immediately available, flexible, and transparent funding with robust systems of accountability powered by national civil society and local committees could support the steady, long-term execution of existing strategies.

True pandemic preparedness and response requires global health actors to accelerate the shift from practices that cause harm to practices that accelerate impact. By dispensing with ways of working that hinder CHW institutionalization and professionalization, resilient health systems are within our grasp. For example, the vast majority of CHWs who deliver malaria drugs, conduct TB directly observed therapy, or accompany HIV patients, do not receive a salary, in contravention of WHO and International Labour Organization guidelines.^42^^,^^43^ Funders must understand the degree to which the success or “cost-effectiveness” of their grantmaking is underpinned by the unremunerated efforts of predominantly poor women of color (i.e., by measuring the percentage of unpaid CHWs who participate in their initiatives), and they must work until that percentage is “zero.”

By dispensing with ways of working that hinder CHW institutionalization and professionalization, resilient health systems are within our grasp.

Table 2 provides additional, though not exhaustive, examples of harmful practices and more promising alternatives for a wide spectrum of funder types and roles.^44^^–^^47^ These examples paint a picture of dynamics that have hindered the current response and can be reversed before the next pandemic.

**TABLE 2.** Considerations for Transforming the Status Quo in Community Health Financing

Community Health Financing Practices That Cause Harm	Community Health Financing Practices That Accelerate Impact
**High transaction costs:** Complexity of donor processes (different funding windows, application deadlines) and documentation requirements. Funding sometimes only reaches communities months or even a full year after an emergency starts Example: For any country's annual budget, multiple donors contribute 10%–12% each toward the budget. These funders have asynchronous funding cycles, documentation requirements, metrics, application lengths, and processes.	**Pooling:** Donors pooling funding not only reduces transaction costs but also facilitates greater alignment and the flexibility to respond to unexpected crises such as pandemics. Aligning grant requirements and templates to reduce application burden would best leverage limited government bandwidth. Example**:** One example of this is the Risk Pool Fund, “a collaborative experiment that makes fast, flexible funding available to pre-selected non-profits that are encountering an unexpected obstacle that threatens impact.”^44^
**Ear-marked and inflexible:** Funding single disease areas (e.g., malaria) independently from the larger health system leads to inefficiency in limited resources Example: In one country, a CHW who has not received payment for months has received 5 smartphones to track disease-specific indicators. Not only is the value of the smartphones higher than the CHW's stipend payment, but the smart apps do not capture all duties, so the CHW has to carry both hard copy tools and the phones.	**Unshackle:** Give unrestricted funding or reduce restrictions on grants. Holding entire programs accountable for their performance is a more rigorous form of accountability than restricting line items. Examine any necessary restrictions for potential negative unintended consequences/spillover effects. Example: Collaborative funding for digital health across multiple technology providers to support pandemic preparedness and digital response from Rockefeller and other philanthropic entities. It appears that this collaborative, rather than competitive, funding model will continue in the wake of the pandemic and could be a strong model for building robust digital health systems. Similarly, several funders committed to providing unrestricted funds during the pandemic—such efforts should broaden and continue.
**Contrary to evidence:** RFPs and grant applications are not consistently designed and evaluated based on evidence-based guidelines like the CHW AIM tool and the WHO CHW guideline. Programs give priority to new or “innovative” approaches, at the expense of tried and true practices known to improve CHW program performance, like fair pay, ongoing training, and dedicated supervision. Example: A large foundation involved in the CHW space funded a vertical (malaria-specific), volunteer-based “project” in a country with an existing professionalized CHW program.	**Evidence-based:** Provide funding based on evidence-based guidelines and tools like CHW AIM to design RFPs and evaluate investment opportunities. Example: In Mali, the Global Fund pays for dedicated CHW supervision, based on government policy and studies published on results in trial sites. While the funding was originally for 1 year, it has now been renewed as part of a larger 3-year national funding package, NFM3.
**Late to pay:** Ineffectively designed mechanisms lead to late payment of CHWs and their supervisors and delayed procurement of the tools they need. This can destroy morale and program effectiveness. Example: We have seen funder lateness, as well as primary recipient (NGO) lateness to pay due to contingencies placed by the funder (for example, a paper report may need to travel from a remote village to the capital before a regular monthly salary is released, leading to months of delay). One author of this article is a CHW working on the COVID-19 vaccination campaign in Malawi where wages have been delayed for 3+ months—a fact he and his colleagues find profoundly demotivating.	**Optimize disbursement:** Optimize, and when needed, redesign disbursement processes to prevent delayed payment and procurement. Example: In Cambodia, a funds-flow analysis helped identify why these bottlenecks are occurring and what actions are needed to prevent them in the future (e.g., development of SOPs, expenditure tracking systems, standardized funds request forms).^45^ In Liberia, mobile money payments to CHWs were instituted to prevent delays. ^46^
**Appropriation of sovereignty:** Funding that does not align with and support national and sub-national government-led strategy can undercut government sovereignty, leadership, and effectiveness. Example: Almost no private philanthropic funders require CSOs/NGOs to (1) prove/substantiate in applications how their plans align and support national strategy, (2) confirm government partnership (e.g., via partnership agreement already signed).	**Align with and support:** government strategy and invest to build up the national system. Example: In 2021, the U.S. President's Malaria Initiative announced the removal of restrictions on the funding of CHW salaries.^47^
**Pressured exploitation:** Funding restrictions have a wide array of unanticipated negative consequences. Multiple major funding agencies will not fund salaries at all, making it difficult for governments to implement global recommendations or to create long-term financing pathways for CHW payment, which often require a mix of international and domestic investment. Example: We have witnessed funders pressure governments and grantees to reduce pay for CHWs and their supervisors to well below minimum wage. Such pressure undermines years of work that went into establishing norms for fair and effective payment of community health workers and makes it much harder for governments to do the right thing (Notably, NGO CEOs are almost never asked to justify the sustainability of having funding agencies paying their salary yet are asked constantly to justify the sustainability plan for CHWs, who are predominantly women living in poverty.)	
**Short-circuiting change:** Short grant and/or financing cycles move grantees toward the easiest strategies for getting funding quickly out the door (such as procuring a large quantity of a given commodity) and away from transformative, high-impact investments Example: While the speed of disbursement is critical and often lacking, duration of commitment is also often lacking. Delayed, slow disbursement of funds, particularly during Ebola 2014 and COVID-19 in 2020–2021, has negatively impacted outbreak response. Relatedly, short-duration commitments create pressure for less effective quick fixes and subvert government efforts to make transformative change in their health care systems.	**Stay:** Longer term, predictable investments support governments and their partners to commit to creating enduring, high-impact community health systems. Longer duration commitments allow governments to invest in recruiting, training, accrediting paid, professionalized CHWs in alignment with WHO guidelines and CHW AIM scorecard. Example: Thomas J. White, cofounder of Partners in Health, funded work in Haiti for his entire life. Institutional funders typically fund in months or years, not decades.

Abbreviations: AIM, Assessment and Improvement Matrix; CEO, chief executive officer; CHW, community health workers; COVID-19, coronavirus disease; CSO, civil society organization; NFM3, new funding model 3; NGO, nongovernmental organization; RFP, request for proposal; SOP, standard operating procedure; WHO, World Health Organization.

## CONCLUSION

The COVID-19 pandemic presents an opportunity for landmark improvements in the ability of health care systems to reach and serve everyone—even in difficult circumstances. CHWs have long played a critical role in preventing, detecting, and responding to pandemics across the globe. To expand, improve, and institutionalize these services, changes in the approach to bi/multilateral aid and private philanthropic investments in low- and middle-income countries are required. First, we must do no harm. Then we must do much more, much better. Practices that hinder CHW stability, support, and labor rights can be intentionally replaced with those that enable sovereign, resilient health systems to flourish. The time is now.

## Notes

**Peer Reviewed**

**First published online:** April 18, 2022.
